# Sublethal effects of contaminants on marine habitat‐forming species: a review and meta‐analysis

**DOI:** 10.1111/brv.12630

**Published:** 2020-07-02

**Authors:** Mariana Mayer‐Pinto, Janine Ledet, Tasman P. Crowe, Emma L. Johnston

**Affiliations:** ^1^ Centre for Marine Scince and Innovation, Evolution & Ecology Research Centre, School of Biological, Earth and Environmental Sciences University of New South Wales Sydney New South Wales 2052 Australia; ^2^ Sydney Institute of Marine Sciences Mosman New South Wales 2088 Australia; ^3^ Earth Institute and School of Biology & Environmental Science, Science Centre West University College Dublin Belfield Dublin 4 Ireland

**Keywords:** biogenic habitats, bivalves, corals, ecosystem functioning, ecosystem services, foundation species, kelp, mangrove, marine systems, metals, PAHs, plants, pollution, saltmarsh, seagrass

## Abstract

Contaminants may affect ecosystem functioning by reducing the fitness of organisms and these impacts may cascade through ecosystems, particularly if the sensitive organisms are also habitat‐forming species. Understanding how sub‐lethal effects of toxicants can affect the quality and functions of biogenic habitats is critical if we are to establish effective guidelines for protecting ecosystems. We carried out a global systematic review and meta‐analysis critically evaluating contaminant effects on properties of habitat‐formers linked to ecosystem functioning. We reviewed a total of 95 publications. However, 40% of publications initially captured by the literature search were identified as having flaws in experimental design and ~11% did not present results in an appropriate way and thus were excluded from the quantitative meta‐analysis. We quantitatively reviewed 410 studies from 46 publications, of which 313 (~76%) were on plants and seaweeds, that is macro‐algae, saltmarsh plants and seagrasses, 58 (~14%) studied corals and 39 (~10%) looked at toxicant impacts on bivalves, with 70% of those on mussels and the remaining studies on oysters. Response variables analysed were photosynthetic efficiency, amount of chlorophyll *a* (as a proxy for primary production) and growth of plants, seaweeds and corals as well as leaf area of plants. We also analysed filtration, growth and respiration rates of bivalves. Our meta‐analysis found that chemical contaminants have a significant negative impact on most of the analysed functional variables, with the exception of the amount of chlorophyll *a*. Metals were the most widely harmful type of contaminant, significantly decreasing photosynthetic efficiency of kelps, leaf area of saltmarsh plants, growth of fucoids, corals and saltmarsh plants and the filtration rates of bivalves. Organic contaminants decreased the photosynthetic efficiency of seagrass, but had no significant effects on bivalve filtration. We did not find significant effects of polycyclic aromatic hydrocarbons on any of the analysed functional variables or habitat‐forming taxa, but this could be due to the low number of studies available. A meta‐regression revealed that relationships between concentrations of metal contaminants and the magnitude of functional responses varied with the type of metal and habitat‐former. Increasing concentrations of contaminants significantly increased the negative effects on the photosynthetic efficiency of habitat‐formers. There was, however, no apparent relationship between ecologically relevant concentrations of metals and effect sizes of photosynthetic efficiency of corals and seaweeds. A qualitative analysis of all relevant studies found slightly different patterns when compared to our quantitative analysis, emphasising the need for studies to meet critical inclusion criteria for meta‐analyses. Our study highlights links between effects of contaminants at lower levels of organisation (i.e. at the biochemical and/or physiological level of individuals) and ecological, large‐scale impacts, through effects on habitat‐forming species. Contaminants can clearly reduce the functioning of many habitat‐forming marine species. We therefore recommend the adoption of routine measures of functional endpoints in monitoring and conservation programs to complement structural measures.

## I. INTRODUCTION

Habitat‐forming species, also known as foundation species (Dayton, 1972), are of great importance in maintaining biodiversity (Bulleri *et al*., 2018) and can modulate ecological and biochemical processes (Ellison *et al*., 2005), many of which are directly linked to the provision of ecosystem services (Christensen *et al*., 1996; DeFries, Foley, & Asner, 2004). Indeed, the very presence of such habitat‐formers is often considered a proxy for a raft of associated biodiversity and ecosystem services (Harding *et al*., 2001). However, anthropogenic impacts on ecosystem functioning can occur not only *via* changes to the abundance and diversity of habitat‐forming species, but also through effects on their physiology, metabolism and/or behaviour (Díaz *et al*., 2007). Biomonitoring strategies for conservation planning and management that focus solely on the abundance of these key species (Harding *et al*., 2001; Phillips & Blackshaw, 2011) are likely to overlook potentially important impacts of stressors on their fitness and function (Mayer‐Pinto *et al*., 2018*b*).

### (1) Sublethal effects of stressors and ecological implications to systems

Stressors can have a range of effects at all levels of biological organisation, from individual organisms and populations to whole systems. The ecological effects of stressors on communities or ecosystems, and their consequences for the functioning of systems and the provision of services are of great concern for managers and conservationists worldwide. However, it is *via* the assessment of stressor impacts at different levels of organisation that we can gain insight into the effects of stress, their mechanistic bases and their potential ecological and evolutionary consequences (Maltby, 1999). The sublethal effects of stressors on individuals can elicit important and harmful ecological impacts on communities and ecosystems. Increases in temperature, for instance, can affect growth rates of corals and cause bleaching, which, in turn, can translate into changes in rates of mortality and fecundity, with long‐term consequences for the overall populations of these organisms (e.g. Michalek‐Wagner & Willis, 2001; Edmunds, 2005). Similarly, long‐term sublethal effects of contaminants on organisms can have significant long‐term ecological impacts on entire systems (Peterson *et al*., 2003). The *Exxon Valdez* oil spill released approximately 41 million litres of crude oil into Prince William Sound, resulting in significantly high local concentrations of polycyclic aromatic hydrocarbons (PAHs) (Bence, Kvenvolden, & Kennicutt Ii, 1996). The spill had devastating effects that lasted more than a decade in the affected systems due both to direct effects of the chronic, toxic persistence of oil (PAHs) and to indirect effects which occurred well beyond the acute‐phase mortality; together these compromised the health, growth and reproduction of several local populations (Peterson *et al*., 2003). Oil exposure indirectly reduced survivorship of salmon, for example, by decreasing the growth rates of individuals, resulting in increased predation [Peterson *et al*., 2003 and references therein]. The lack of consideration of sublethal effects can significantly underestimate the effects of stressors on communities and systems, potentially leading to disastrous regulatory and management actions (Desneux, Decourtye, & Delpuech, 2007). A further example is the use of pesticides, such as pyrethroids, where documented sublethal effects on several species were not considered in an integrated pest‐management context, causing many ‘unexpected’ impacts on populations and communities such as amphibians and arthropods (Desneux *et al*., 2007; Relyea & Diecks, 2008). Moreover, if contaminants impact key species, such as habitat‐forming species, then the overall consequences for communities and systems can be expected to be even more substantial.

### (2) Habitat‐forming species

Corals, seaweeds, plants and bivalves such as oysters are common habitat‐formers in marine systems, which are among the most diverse and valuable habitats on the planet (Costanza *et al*., 1997, 2014). It has been estimated that marine habitats, from the intertidal zone to open ocean systems, provide over US$ 420 K per ha per year through the provision of services such as food, trade and recreational opportunities (Costanza *et al*., 2014). For example, each hectare of seagrass provides an estimated US$ 34 K per annum of services such as coastal protection from storms, support of commercial fisheries and nutrient cycling (Short *et al*., 2011; Costanza *et al*., 2014). The consequences of the absolute loss of these habitat‐forming organisms to system functionality, and therefore, to the provision of ecosystem services, are relatively well known (Duarte, 2002; Hughes *et al*., 2003; Wernberg *et al*., 2016). However, impacts on the functioning of ecosystems can also occur through sublethal changes in the physiological performance of organisms (e.g. filtration rates and growth) that are involved in the maintenance of such functions (Mumby *et al*., 2011; Johnston & Mayer‐Pinto, 2015; Montalto *et al*., 2016; Mayer‐Pinto *et al*., 2018*a*). Biological filtration removes particles and cells of phyto‐ and microbial plankton from the water column in a process that accelerates mineralisation of organic substances and ‘purifies’ the water‐column (Ostroumov & Widdows, 2006). Many habitat‐forming bivalves (i.e. oysters, mussels) are the dominant filter feeders in the habitats they form and, therefore, the rate at which they feed and grow can be used to estimate the overall rates of clearance (or filtration) and productivity of these habitats (Beck *et al*., 2011). Changes in the filtration rates of reef‐forming bivalves can, in turn, affect local water quality with many potential consequences on the associated biodiversity. Similarly, the photosynthetic activity of habitat‐forming macro‐algae makes a substantial contribution to the primary productivity of the ecosystem, with Atlantic kelp, including species such as *Saccharina latissima* and *Laminaria digitata*, for example, contributing gross primary production of 1 kg C m^−2^ year^−1^ (Mann, 1982; Smale *et al*., 2013). Habitat‐forming macro‐algae such as kelps can provide up to 90% of total carbon to coastal food webs (Duggins, Simenstad, & Estes, 1989). Changes in the physiological performance of habitat‐formers induced by contaminant stress can therefore have important consequences for the overall functioning of ecosystems.

### (3) Chemical contaminants

Chemical contaminants are global drivers of change across multiple spatial scales and are increasingly ubiquitous in ‘natural’ and urban systems, being found even in the most remote and inaccessible areas of the globe (Jamieson *et al*., 2017). In the United States alone, there are more than 80000 registered chemicals (GAO, 2013), each with different chemical properties that have the potential to affect physiological and ecological traits of a wide range of organisms (e.g. Bryan, 1971; McCahon & Pascoe, 1990). The extensive and rapid growth of human populations in close proximity to waterways has led to increasing amounts and numbers of contaminants such as metals, pesticides, PAHs and organic compounds in coastal environments. In urbanised or industrialised areas, there are many pathways by which chemical contaminants enter coastal habitats, such as non‐point‐source runoff from developments and waste dumping into estuaries or nearshore environments (GESAMP, 1982; Kennish, 2002; Johnston & Mayer‐Pinto, 2015). Agricultural and boating activities are other important sources of coastal water pollution. Agriculture is responsible for the application of millions of tons of fertilisers and pesticides to crops each year (FAO, 2006; Schwarzenbach *et al*., 2006), which are washed or blown away from target areas and eventually discharged into rivers, groundwater and marine systems (Jones, 2005; Johnston & Mayer‐Pinto, 2015). Shipping and boat activities have been identified as one of the main causes of pollution in places often assumed to be protected (e.g. *Antarctica*) (Negri & Marshall, 2009) and are often associated with antifouling paints (Dafforn, Lewis, & Johnston, 2011). Antifouling measures to avoid the colonisation of organisms on hulls of boats and ships have traditionally used paints with added contaminants such as copper, zinc and tributyltin that gradually leach, resulting in high levels of contamination in the environment (Dafforn *et al*., 2011). Furthermore, vessel mooring facilities such as ports and marinas are associated with increased concentrations of metals and PAHs. Each of these types of contaminant have different modes of action. Metals, for example, bind with important enzymes and proteins, changing their ability to function properly and causing malfunctioning or death of cells (Bryan, 1971) while organic compounds can disrupt the endocrine, reproductive and immune systems of organisms (Porte *et al*., 2006). Some types of pesticide can disrupt photosystem II (PSII) in primary producers, affecting their photosynthetic capacity (Jones, 2005). Therefore, these toxicants can affect individuals and populations in various ways, potentially leading to changes in biodiversity and ecosystem functioning (Johnston & Roberts, 2009; Johnston, Mayer‐Pinto, & Crowe, 2015). The magnitude and direction of such changes are, however, dependent on the environmental context, including the species or attributes/traits affected, the type of contaminant and its concentration as well as the period/intensity of exposure (i.e. for how long and at what concentration were species/communities exposed to the contaminant). For instance, some species or functional traits may be tolerant to some contaminants, but not to others. Furthermore, different stressors or toxic contaminants can have positive, negative or interactive effects on individual functions (Alsterberg, Sundbäck, & Gamfeldt, 2014). Finally, the magnitude of impacts is posited to be directly and positively linked to the concentration of the contaminant and period of exposure, but experimental results within and among studies are mixed (e.g. Márquez‐García *et al*., 2013; Peters, Bundschuh, & Schäfer, 2013; Cambrollé *et al*., 2016) and likely to vary depending on the presence of other contaminants or stressors (Schiedek *et al*., 2007; Relyea, 2009; O'Brien *et al*., 2019).

### (4) Direct and indirect effects of contaminants

Chemical contamination can potentially alter the functioning of systems through multiple pathways. Direct effects include increased mortality of sensitive species, leading to lower overall biodiversity, which in turn, is linked to decreases in functioning (e.g. Hooper *et al*., 2005; Cardinale *et al*., 2012; Hooper *et al*., 2012). Contaminants are linked to decreases in global biodiversity (Johnston & Roberts, 2009) and in a qualitative systematic review on contaminant effects on ecosystem functioning, Johnston *et al*. (2015) found that these toxicants generally altered functions of marine systems by reducing overall productivity and increasing respiration. Their review, however, was based on functional measurements linked to whole trophic groups (e.g. phytoplankton or benthic organisms) or systems (e.g. multiple trophic components of any given system) and did not identify pathways of change. Nevertheless, indirect effects, such as those mentioned below, can be as important as direct effects in structuring communities and systems, and increasing evidence has shown that indirect effects of several chemical contaminants, such as pesticides, are more common and complex than their direct effects (Fleeger, Carman, & Nisbet, 2003; Rohr, Kerby, & Sih, 2006; Relyea & Diecks, 2008; Rohr *et al*., 2008; Clements & Rohr, 2009; Mayer‐Pinto, 2017).

Species in a community often interact with each other, but the outcome of such interactions might depend on the attributes of the specific individuals, species or functional groups involved (Vellend & Geber, 2005; Marzinelli *et al*., 2012). For instance, whether the species affected by contaminants are habitat‐formers or highly mobile epibiota is likely to influence the ultimate effects on the overall function of the systems, regardless of possible losses in biodiversity (McMahon *et al*., 2012). Where single species have large effects on their communities, such as keystone species or habitat‐formers, changes in their physiological, metabolic or behavioural traits have the potential to alter and/or shape the interactions of whole collections of species – including those not directly involving the habitat‐forming species itself (Ellison *et al*., 2005). For example, some aggressive, territorial individuals of the limpet *Lottia gigantea* can keep large areas of rocky shore free of other herbivores, resulting in refugia for micro‐algae and newly recruited macrophytes (Stimson, 1970). Trophic cascades are arguably one of the most common types of indirect impact linked to changes driven by chemical contaminants. Examples include effects on the behaviour and/or abundance of predators/grazers, resulting in increases in the abundance of prey or primary producers (Fleeger *et al*., 2003; Evans‐White & Lamberti, 2009; Saaristo *et al*., 2018), with direct implications for the overall primary productivity of systems. Additionally, contamination is highly likely to affect habitat quality (Roberts, Johnston, & Poore, 2008b), which is an important determinant of species occupancy (e.g. Wiegand *et al*., 2008; Rubene, Wikars, & Ranius, 2014). Brown bears (*Ursus arctos*)’ selection of habitats, for instance, is linked to the functioning of systems, e.g. primary productivity rather than vegetation structure (Wiegand *et al*., 2008). Similarly, many species of herbivores are associated, to a greater degree, with rapidly growing species of seagrass than with slow‐growing ones (Mariani & Alcoverro, 1999; Burkholder, Heithaus, & Fourqurean, 2012). Understanding the effects of contaminants on the functional attributes of habitat‐forming species is therefore critical if we are to evaluate the full consequences of anthropogenic contamination on ecosystems.

### (5) Associated biodiversity

Although contamination of any given place has the potential to affect all organisms living in the area, highly mobile species, such as fish and some invertebrate grazers, may be able to move to other, more suitable, non‐contaminated areas (Roberts, Poore, & Johnston, 2007; Roberts *et al*., 2008*a*; Tierney, 2016). Habitat‐forming species are, however, usually sessile by nature, hence contamination impacts on these organisms are likely to be longer term and can persist even after the source of pollution has been eliminated (Perrett, Johnston, & Poore, 2006). Importantly, contaminant impacts on habitat‐formers can directly affect the associated faunal and floral communities long after local contamination has ceased, for example through impacts on their re‐colonisation and recovery (reviewed by Roberts *et al*., 2008*b*). For instance, *in situ* recruitment of epifaunal organisms, such as amphipods and gastropods, is reduced on macroalgae experimentally contaminated with copper when compared to uncontaminated algae (Roberts, Poore, & Johnston, 2006). In addition, complete mortality of grazing gastropods was observed within 1–4 weeks of continuous dietary exposure to contaminated algae (Weis & Weis, 1992). Similarly, carnivorous gastropods avoided eating metal‐contaminated oysters, resulting in lower growth rates compared to gastropods fed with ‘uncontaminated’ oysters (Weis & Weis, 1993). Therefore, as concluded by Roberts *et al*. (2008*b*) contamination of biogenic habitats can have direct impacts on the survival and fitness of their associated fauna and flora. Moreover, research has shown that whilst the abundance and richness of epifaunal organisms and other communities associated with habitat‐formers, such as seagrass meadows, partly recovered prior to the full recovery of seagrass, a complete recovery of the habitat‐former was required before the epifaunal community matched that of the natural seagrass meadow (McSkimming *et al*., 2016). The assessment of sub‐lethal effects of contaminants on habitat‐formers may therefore help predict potential impacts on whole communities and systems.

### (6) Ecotoxicology and functional impacts

Although traditional ecotoxicology research has long recognised that contamination can have important sublethal effects on organisms by altering their physiological performance (e.g. growth and primary productivity) (McLusky, Bryant, & Campbell, 1986; Connan & Stengel, 2011), such studies very rarely interpret their findings within an ecosystem function context (Johnston *et al*., 2015). By contrast, ecological studies assessing functional impacts of stressors usually focus on the detrimental effects mediated by structural changes such as biodiversity loss (Cardinale *et al*., 2006; Cardinale *et al*., 2012; Byrnes *et al*., 2014), thus neglecting to consider other, potentially stronger, impacts on ecosystem functioning that may occur without the loss of species or traits, for example *via* sublethal effects. In fact, ecologists in general have long overlooked the role of contaminants as agents of global change (Bernhardt, Rosi, & Gessner, 2017).

To examine whether general patterns exist in the functional consequences of chemical contaminants on marine habitat‐forming species, we carried out a global systematic review and a series of meta‐analyses. Recent developments in the field of meta‐analysis now allow the integration of complex data with multiple layers, such as different measurements, concentrations of contaminants and species/functional groups, in order to identify overall patterns and to evaluate consistency among study findings (Nakagawa & Santos, 2012; Hedges & Olkin, 2014).

Specifically, we asked: (*i*) with which taxa and types of contaminants has research examined responses of physiological functions to non‐nutrient contamination; (*ii*) how were the studies designed (e.g. regarding controls, replication, field *versus* laboratory studies, etc.); (*iii*) what are the physiological functions that are most affected; and (*iv*) do the effects of contaminants vary according to the type of contaminant and/or habitat‐formers involved and if so, what are the most influential contaminants and the most vulnerable habitat‐formers.

## II. METHODS

### (1) Literature search

**Fig 1:**
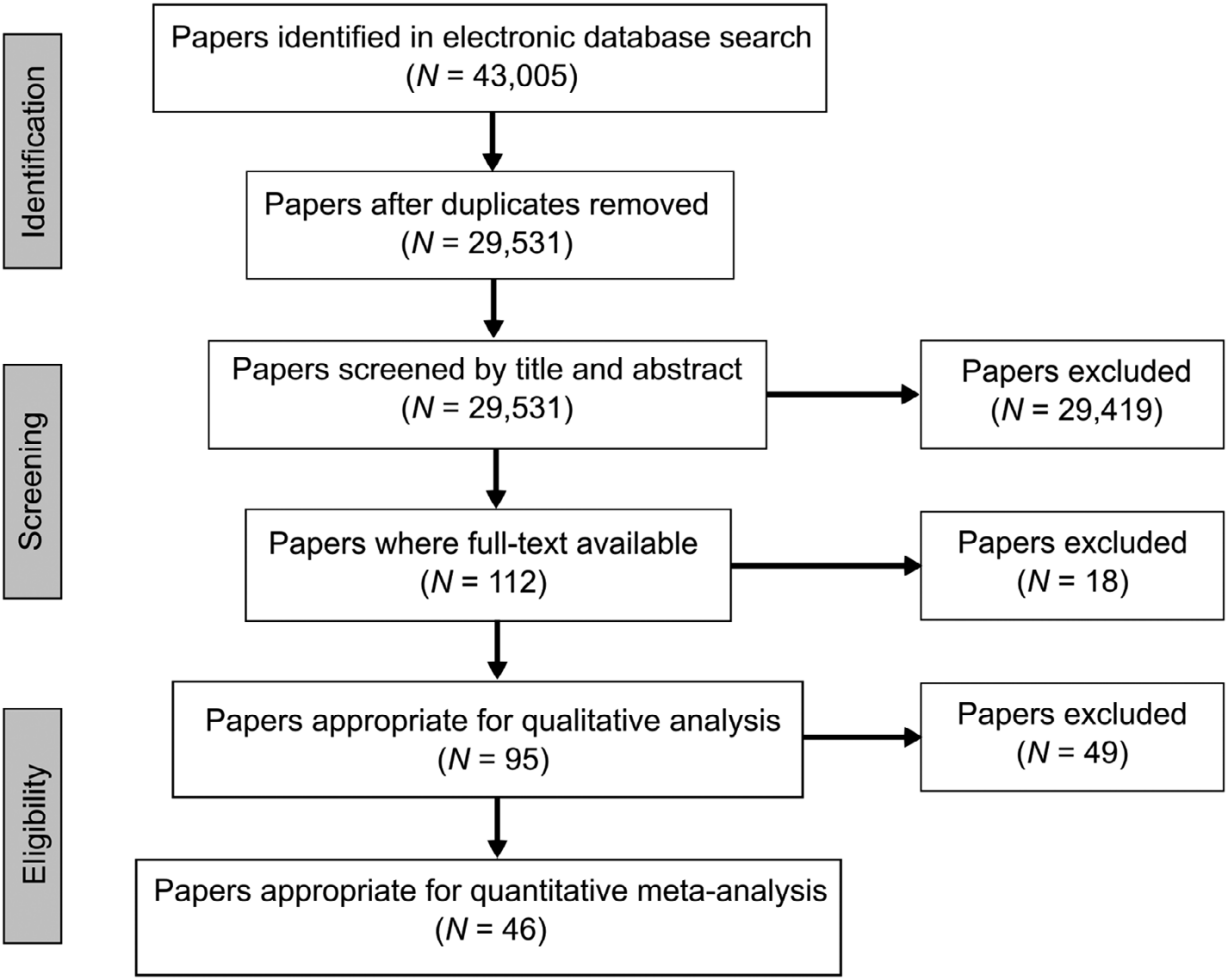
PRISMA diagram for the identification of literature used in the qualitative assessment and quantitative meta‐analysis of functional responses of habitat‐forming species to contamination. Details of search terms are provided in Section II.1.

We systematically reviewed published studies on the effects of chemical contaminants on habitat‐forming species, using a structured search through the online search engines *ISI Web of Science* and *Current Contents Connect*. The search used the key words brown alga*, kelp*, mangrove*, salt marsh*, seagrass*, bivalve* and coral* in combination with respiration, respir*, productivity, product*, photosynthesis, clearance rate*, filter*, growth and purification and contamina* and pollut*. The key words chosen were based on those used by Johnston & Roberts (2009) and Johnston *et al*. (2015), and allowed the inclusion of studies that used broader generic terms, such as ‘oil spills’. We also examined the citation lists of relevant papers identified by this search in order to capture studies that were not included in the initial searches or that had been published in journals not indexed in the databases we searched. The last search date was July 2017. Our search generated 29531 unique papers (Fig. 1).

### (2) Inclusion and exclusion criteria

Studies selected for the review included a comparison among contaminated and non‐contaminated (control) treatments. Only chemical toxicants were considered in this review. Similarly, only physiological aspects of the habitat‐formers that could be directly linked to a particular function, and/or quality, of habitats were included. Therefore, studies solely investigating the impacts of contaminants on the abundance and/or behaviour of species or their cellular/molecular structure were not included, nor were those that only considered bio‐accumulation. As a result, a total of 95 publications were selected for review (Fig. 1).

Each selected study was classified according to: (*i*) type of contaminant [e.g. metals, herbicides, hydrocarbons; based on Johnston & Roberts, 2009 and Johnston *et al*., 2015]; (*ii*) response variable (e.g. photosynthetic activity, filtration rates); (*iii*) organisms (e.g. corals, seaweeds, bivalves); (*iv*) study setting and type – whether it was done in the laboratory or in the field and, in the latter case, whether it involved experimental manipulation or was observational (i.e. a survey or measurement made *in situ*, with no manipulation); and (*v*) appropriateness of design, which was classified as in Johnston *et al*. (2015), using the framework developed by Lyons *et al*. (2012). For bivalves, only reef‐forming species were included in the review. From the systematic review, 46 papers were suitable for inclusion in the meta‐analyses after exclusion of papers with poorly designed experiments, and/or where relevant data were not presented either in text, tables, or graphs (Fig. 1; see online Supporting information, Table S1).

### (3) Quantitative analysis: meta‐analysis effect‐size calculation and moderators

We calculated the size of the effect of contaminants on each type of physiological response (e.g. filtration rates) for all studies that reported means, S.D.s or S.E.s and sample sizes or from which these data could be extracted from figures. Data were extracted from graphs using WebPlotDigitaliser (https://automeris.io/WebPlotDigitizer/). If the data were reported as a time series, we used the data from the final sampling period only (Strain *et al*., 2014). If data were reported on multiple species, different types of contaminants, or at different sites in the same publication, we recorded all information. Some individual publications therefore contributed considerable numbers of studies to the analyses (e.g. if, within a publication, authors assessed the effects of copper on photosynthetic efficiency of three species of seaweeds, this publication contributed a total of three studies to our analyses).

We completed meta‐analyses using the R package metafor (Viechtbauer, 2010) in R gui 3.1.1 (R Core Team, 2016). Effect sizes were calculated using the Hedges’ *g* standardised mean difference (SMD; Borenstein *et al*., 2009) for all functions except filtration rate, due to the large quantity of zeros or negative values, or lack of convergence (in the case of chlorophyll *a*). Effect sizes for filtration rate were calculated using the log ratio of means (ROM in metafor R package). To account for non‐independence in the data, publication identity and study identity, nested in publication, were included as random factors (Noble *et al*., 2017). We checked for publication bias using qualitative tests (funnel plots). We then explored the drivers that we hypothesised would moderate the magnitude and direction of effects of contaminants on the functional attributes of habitat‐formers, which included the type of contaminant and organisms. To assess the overall effects of contaminants (regardless of type) on each category of organism (e.g. primary producers or invertebrates), no fixed moderators were included in the models. If, when including fixed moderators, the number of studies was less than 3 (i.e. *N* ≤ 3), these were not analysed. They were, however, included in the analyses to assess the overall effects of contaminants (i.e. with no fixed moderators). Where concentrations of the same contaminant were varied within a single study, with one shared control for two or more treatments, we created a variance–covariance matrix to take into account within‐study correlated variances (Olkin & Gleser, 2009; Noble *et al*., 2017). Matrices were calculated for each model according to the effect size used (i.e. Hedges’ *g*). To assess relationships between effect sizes and additional moderator variables obtained during data extraction, such as type of contaminant and their concentration and time of exposure, multiple meta‐regressions were carried out using the function mareg from the R package MAd (Del Re & Hoyt, 2014). Because concentrations of contaminants were measured using different units (e.g. μg l^−1^, μmol l^−1^) across studies, meta‐regressions were performed for each specific unit of measurement and were focused on the group of contaminants for which we had the most data: metals. We also carried out two sets of meta‐regressions, one with all the metal concentrations used in the selected studies, and a second limiting the analysis to a more environmentally relevant range of concentrations (i.e. including only concentrations up to 500 μg l^−1^ from all analysed studies).

### (4) Qualitative analysis

To obtain a more holistic picture of the functional consequences of contaminants on habitat‐forming species, we also carried out a critical qualitative review of all the information available, including poorly designed studies or those that did not present data in the appropriate format for the meta‐analyses. Publications were analysed for general effects of contamination on habitat‐former functions, either recorded as an increased effect, decreased effect, or no effect. For publications that reported multiple concentrations of contaminant effects on habitat‐formers, only the result of the highest concentration was reported (as per Johnston *et al*., 2015). Therefore, although the total number of publications used in the qualitative review is greater than the number of publications used in the meta‐analysis, the number of studies (i.e. pair‐wise comparisons within each publication) used in the qualitative analyses is smaller. To assess the influence of poor experimental design on overall conclusions, we analysed all papers qualitatively, based on inferences drawn by the authors regardless of the appropriateness of the design. We then discussed these results with findings from the meta‐analysis (quantitative), which only included well‐designed studies.

## III. RESULTS

### (1) Summary information

We qualitatively reviewed 243 studies from the 95 publications that met our criteria, which were largely from temperate systems (especially Europe and North America). Papers covered 81 species, which were broadly classified into bivalves, corals, and plants and seaweeds. Plants included mangroves, saltmarsh plants and seagrasses, while seaweeds included fucoids and kelps. Plants and seaweeds made up 53% of the studies analysed (*N* = 129), followed by 25% on corals (62), and 21% on bivalves (52). Selected studies covered the effects of 100 unique contaminants across all habitat‐forming groups. Metals, in particular copper, were the most studied chemical contaminant, with a total of 85 studies (35%), followed by hydrocarbons and herbicides with 51 studies each (~21%). The concentrations of contaminants varied greatly across studies. Of the physiological variables ~45% of the studies (110) evaluated the effects of chemical contaminants on the photosynthetic efficiency of habitat‐formers and ~ 42% (101) looked at growth rates. Approximately 88% of studies were laboratory‐based experiments (213). 98 studies from a total of 38 publications analysed (~40% of all publications) had problems with their design (e.g. poor replication, lack of proper controls, or both), or had unclear methodology; Fig. S1), while 30 studies from 11 publications (~11%) did not have data reported appropriately for subsequent quantitative analyses.

For the quantitative part of the review, that is the meta‐analyses, we analysed a total of 410 studies (pair‐wise comparisons) from the 46 selected publications (Table S2). Of those, approximately 76% (313) were on plants and seaweeds, of which 45% (142) were on saltmarsh plants. The effects of contaminants on corals were evaluated on ~14% of studies (58), while only 10% (39) looked at toxicant impacts on bivalves, with 70% of those on mussels and the remaining studies on oysters (Table S2). Therefore, mussels and oysters were grouped together as bivalves. For each research question we report the quantitative results of the meta‐analysis and then compare how the inclusion criteria (appropriately designed studies) altered overall patterns or trends when compared with the qualitative analysis.

### (2) Quantitative review: meta‐analysis

#### (a) *Impacts of contaminants on functional variables of habitat‐formers*

Response variables analysed were photosynthetic efficiency, amount of chlorophyll *a* (as a proxy for primary production; see Johnston *et al*., 2015) and growth of plants, seaweeds and corals. We also analysed leaf area of plants and filtration and respiration rates of bivalves. Most studies on growth rates of bivalves had methodological flaws and/or were not reported appropriately and, therefore could not be included in the quantitative analysis (but see Section III.4).

**Fig 2:**
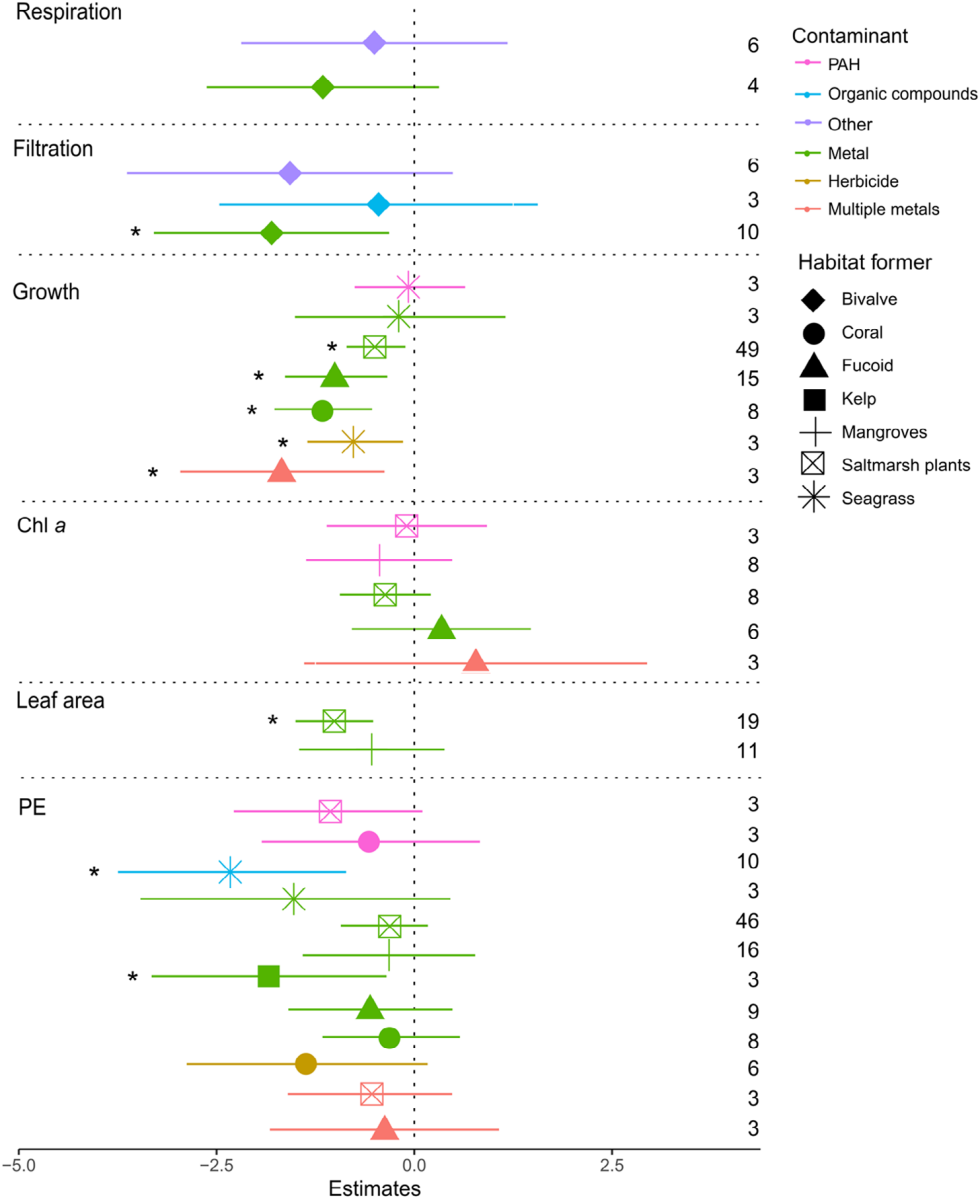
Meta‐analysis results represented as a forest plot with the effects of each contaminant (symbol colour) on specific habitat‐formers (symbol shape). Chl *a*, chlorophyll *a*; PAH, polycyclic aromatic hydrocarbon; PE, photosynthetic efficiency. All data are mean effect sizes with 95% lower and upper confidence intervals for each effect. An effect size of zero is indicated by the dotted line). Significant effects are denoted with an asterisk (*). The number of studies (inclusive of all contaminant concentration levels within the study) that contributed to the effect is shown to the right of the plot. Effect sizes derived from *N* < 3 are not shown. Note that effects of contaminants on growth were not analysed for bivalves.

As predicted, the meta‐analyses showed that chemical contaminants have a significant negative impact on many of the analysed functional variables, with exception of the amount of chlorophyll *a* (Fig. 2; Table 1). Metals seem to be the most widely harmful type of contaminant negatively affecting a wide range of variables and types of habitat‐formers (Fig. 2). The susceptibility of each habitat‐forming taxon (i.e. corals, fucoids, seagrasses, etc.) varied according to the function analysed. For instance, the growth of fucoids was more susceptible to metals than that of seagrass (Fig. 2). Interestingly, however, contamination did not affect the concentration of chlorophyll *a* nor the photosynthetic efficiency of saltmarsh plants (Fig. 2). When looking at the effects of metals on the leaf area of plants – a measurement often directly linked with productivity in terrestrial studies – we found that saltmarsh plants were significantly more affected by these toxicants than mangroves (Fig. 2; Table 1). Growth rates of saltmarsh plants, fucoids and corals were significantly reduced by metals while seagrass growth was only impacted by herbicides (Fig. 2). Photosynthetic efficiency of kelps and seagrass was significantly reduced by metals and organic contaminants, respectively. The filtration rate of bivalves was also significantly reduced when these organisms were exposed to metals, but there were no effects of contaminants on respiration rates (Fig. 2). We did not find significant effects of PAHs on any of the analysed functional variables or habitat‐forming taxa. However, it is important to note that the number of studies on PAHs (i.e. the overall replication) was low (maximum *N* = 3 within each group analysed, except for concentration of chlorophyll *a* in mangroves, where *N* = 8; Fig. 2).

**Table 1:** Model coefficients (mean effect size; ES) and 95% lower and upper confidence intervals (L.CI and U.CI, respectively) for functions with habitat‐forming group and contaminant type as moderators

Moderators	Chl *a* (SMD)			PE (SMD)			Growth (SMD)			Leaf area (SMD)			Filtration (ROM)			Respiration (SMD)		
	ES	L. CI	U. CI	ES	L. CI	U. CI	ES	L. CI	U. CI	ES	L. CI	U. CI	ES	L. CI	U. CL	ES	L. CI	U. CL
Coral*metals	—	—	—	−0.28	−1.15	0.59	−1.15***	−**1.77**	−**0.53**	—	—	—	—	—	—	—	—	—
Coral*herbicide	—	—	—	−1.34	−2.86	0.18	—	—	—	—	—	—	—	—	—	—	—	—
Coral*PAH	—	—	—	−0.55	−1.93	0.83	—	—	—	—	—	—	—	—	—	—	—	—
Fucoid*mult. Metals	0.78	−1.39	2.94	−0.38	−1.83	1.07	−1.67*	−**2.96**	−**0.38**	—	—	—	—	—	—	—	—	—
Fucoid*metals	0.34	−0.79	1.47	−0.54	−1.58	0.50	−0.99**	−**1.64**	−**0.34**	—	—	—	—	—	—	—	—	—
Kelp*metals	—	—	—	−1.84*	−**3.32**	−**0.35**	—	—	—	—	—	—	—	—	—	—	—	—
Seagrass*herbicide	—	—	—	—	—	—	−0.75*	−**1.35**	−**0.14**	—	—	—	—	—	—	—	—	—
Seagrass*metal	—	—	—	−1.50	−3.46	0.46	−0.18	−1.51	1.15	—	—	—	—	—	—	—	—	—
Seagrass*PAH	—	—	—	—	—	—	0.06	−0.75	0.64	—	—	—	—	—	—	—	—	—
Seagrass*organic	—	—	—	−2.30**	−**3.75**	−**0.86**	—	—	—	—	—	—	—	—	—	—	—	—
Mangroves*PAH	−0.44	−1.37	0.48	—	—	—	—	—	—	—	—	—	—	—	—	—	—	—
Mangroves*metals	—	—	—	−0.32	−1.41	0.77	—	—	—	−0.54	−1.46	0.38	—	—	—	—	—	—
Saltmarsh*mult. Metals	—	—	—	−0.55	−1.59	0.49	—	—	—	—	—	—	—	—	—	—	—	—
Saltmarsh*metals	−0.37	−0.94	0.21	−0.38	−0.93	0.17	−0.49**	−**0.86**	−**0.11**	−1.01***	−**1.50**	−**0.52**	—	—	—	—	—	—
Saltmarsh*PAH	−0.09	−1.11	0.92	−1.09	−2.28	0.10	—	—	—	—	—	—	—	—	—	—	—	—
Bivalve*metal	—	—	—	—	—	—	—	—	—	—	—	—	−1.81*	−3.29	−0.32	−1.16	−2.62	0.312
Bivalve*organic	—	—	—	—	—	—	—	—	—	—	—	—	−0.45	−2.47	1.56	—	—	—
Bivalve*other	—	—	—	—	—	—	—	—	—	—	—	—	−1.57	−3.63	0.49	−0.51	−2.19	1.178

Credible intervals of the model that do not overlap zero are in bold (*** *P* < 0.0001; ** *P* < 0.001; * *P* < 0.01). Studies on the effects of two or more metals in combination (e.g. Cu and Zn) were classified as multiple metals (mult.metals), while studies that investigated only one type of metal (e.g. Cu or Zn) were classified as metals. Chl a, chlorophyll a; PAH, polycyclic aromatic hydrocarbons; PE, photosynthetic efficiency; SMD, standardised mean difference.

### (3) Meta‐regression analyses of effects of moderator variables

#### (a) *Concentration of metals and effect size*

**Fig 3:**
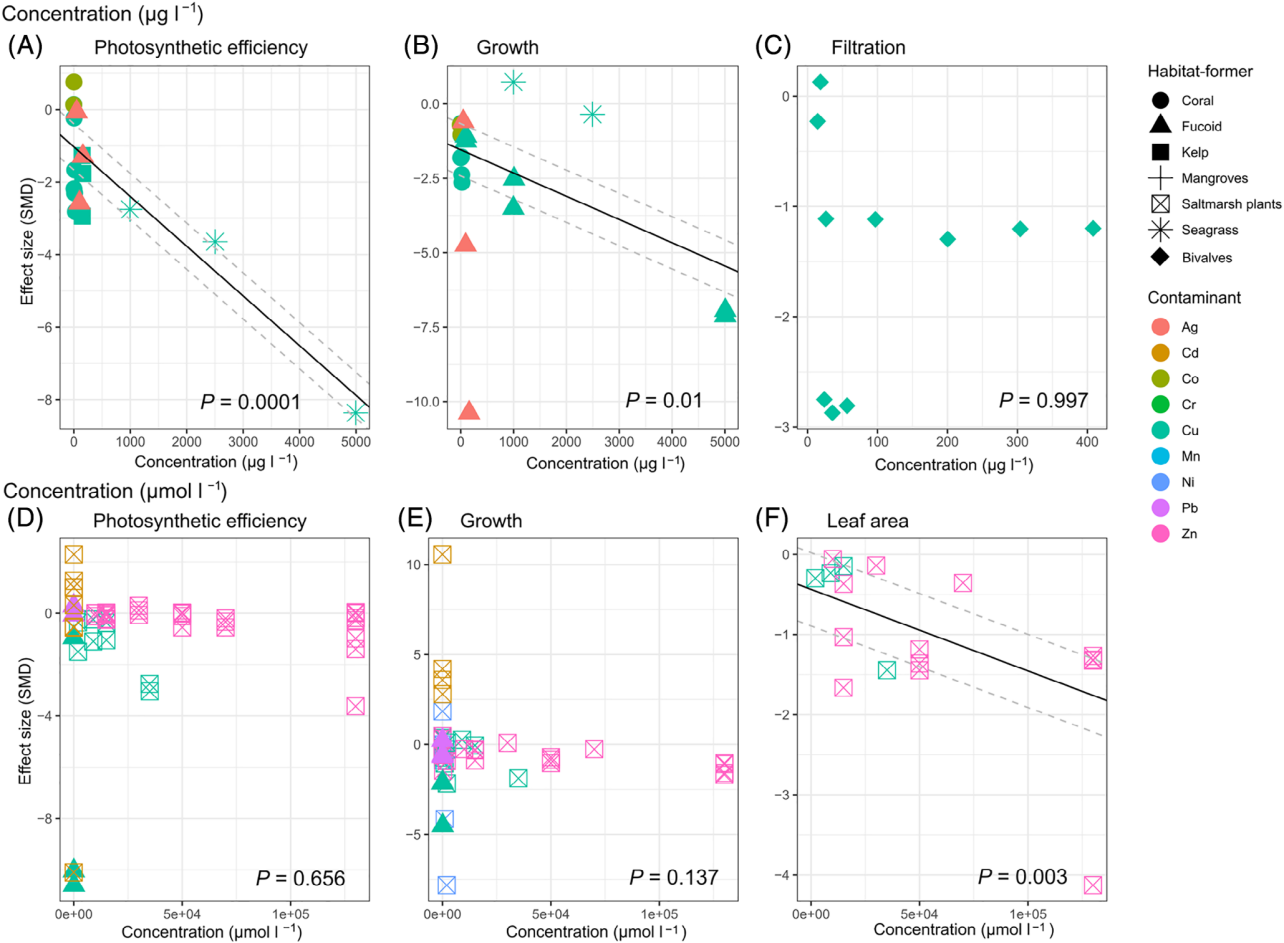
Meta‐regression of effect size (standardised mean difference; SMD) from studies reporting sublethal effects of metals on habitat‐formers at (A–C) increasing concentrations measured in μg l^−1^ and (D–F) increasing concentrations measured in μmol l^−1^. Within function plots, metal contaminants are denoted by symbol colour and types of habitat‐formers by symbol shape, with the calculated model linear regression (when statistically significant; *P* < 0.05) for all data shown in the plot (black line) with 95% confidence limits (grey dashed lines).

**Fig 4:**
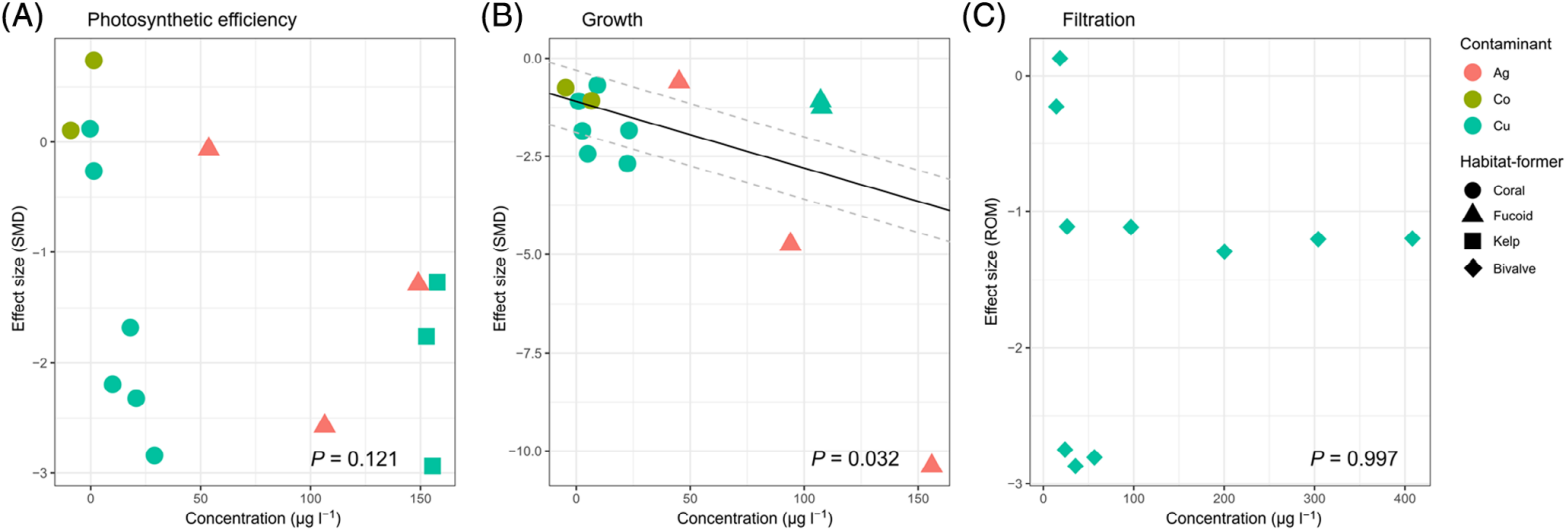
Meta‐regression of effect size (standardised mean difference; SMD) from studies reporting the sublethal effects of silver, copper and cobalt (measured as μg l^−1^) at ecologically relevant concentrations (<500 μg l^−1^) on habitat‐formers. Data points show the tested contaminant (denoted by symbol colour) and habitat‐former (denoted by symbol shape). Linear regression lines are shown in black when statistically significant (*P* < 0.05) with upper and lower confidence intervals as grey dashed lines.

**Fig 5:**
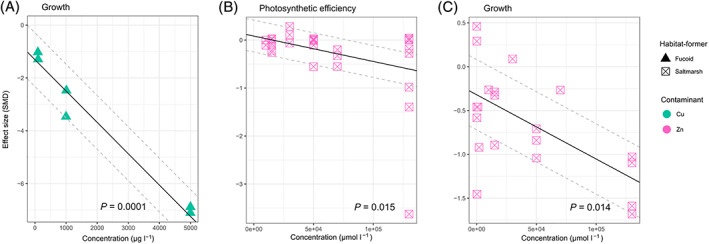
Meta‐regression of effect size (standardised mean difference; SMD) from studies reporting the sublethal effects of copper (μg l^−1^) and zinc (μmol l^−1^) on fucoids and saltmarsh plants. Data points show the tested contaminant (denoted by symbol colour) and habitat‐former (denoted by symbol shape). Linear regression lines are shown in black when statistically significant (*P* < 0.05) with 95% upper and lower confidence intervals as grey dashed lines.

Relationships between metal concentrations and responses of functional variables varied with the type of metal and habitat‐former (Fig. 3). Copper and zinc were, in general, the most studied types of metals (Fig. 3). Increasing concentrations of contaminants (measured in μg l^−1^) significantly increased the negative effects on the photosynthetic efficiency of habitat‐formers (Fig. 3A), but this was driven mainly by effects of copper on seagrass and/or the high concentrations of this metal (Fig. 4A). There was no significant relationship between effect sizes and concentrations of silver, copper and cobalt at ecologically relevant concentrations on the photosynthetic efficiency of corals and seaweeds (Fig. 4A). Similarly, we found no significant relationship between increasing concentrations of contaminants (Cu, Zn, Cd and Pb in μmol l^−1^) and impacts on photosynthetic efficiency of fucoids and saltmarsh plants (Fig. 3D). However, increasing zinc concentrations (μmol l^−1^) significantly increased negative effects on the photosynthetic efficiency of saltmarsh plants (Fig. 5B). There was a significant relationship between increasing concentrations of copper, cobalt and silver (measured in μg l^−1^) and effect sizes of growth of fucoids, corals and seagrass (Fig. 3B). Specifically, we found significant relationships between increasing concentrations of copper and zinc and impacts on the growth of fucoids and saltmarsh plants, respectively (Fig. 5A, C). Increasing concentrations of zinc and copper also significantly increased impacts on the leaf area of saltmarsh plants (Fig. 3F). Interestingly, we found no relationship between concentrations of copper and the effect size for filtration rates of bivalves (Fig. 3C).

We also analysed a subset of studies that measured the effect of metals on habitat‐formers within what we considered ‘environmentally relevant’ levels of contamination, as metal concentrations reported in heavily contaminated marine waters are generally <500 μg l^−1^ (14 studies on photosynthetic efficiency and 13 on growth). With these environmentally relevant concentrations of metals, we found no relationship between concentration of these contaminants and impacts on photosynthetic efficiency of habitat‐formers (Fig. 4A). For the growth of fucoids and corals, however, we found a significant relationship between effect size and increasing concentrations of silver, cobalt and copper up to 150 μg l^−1^ (Fig. 4B).

#### (b) *Duration of exposure to metals and effect size*

**Fig 6:**
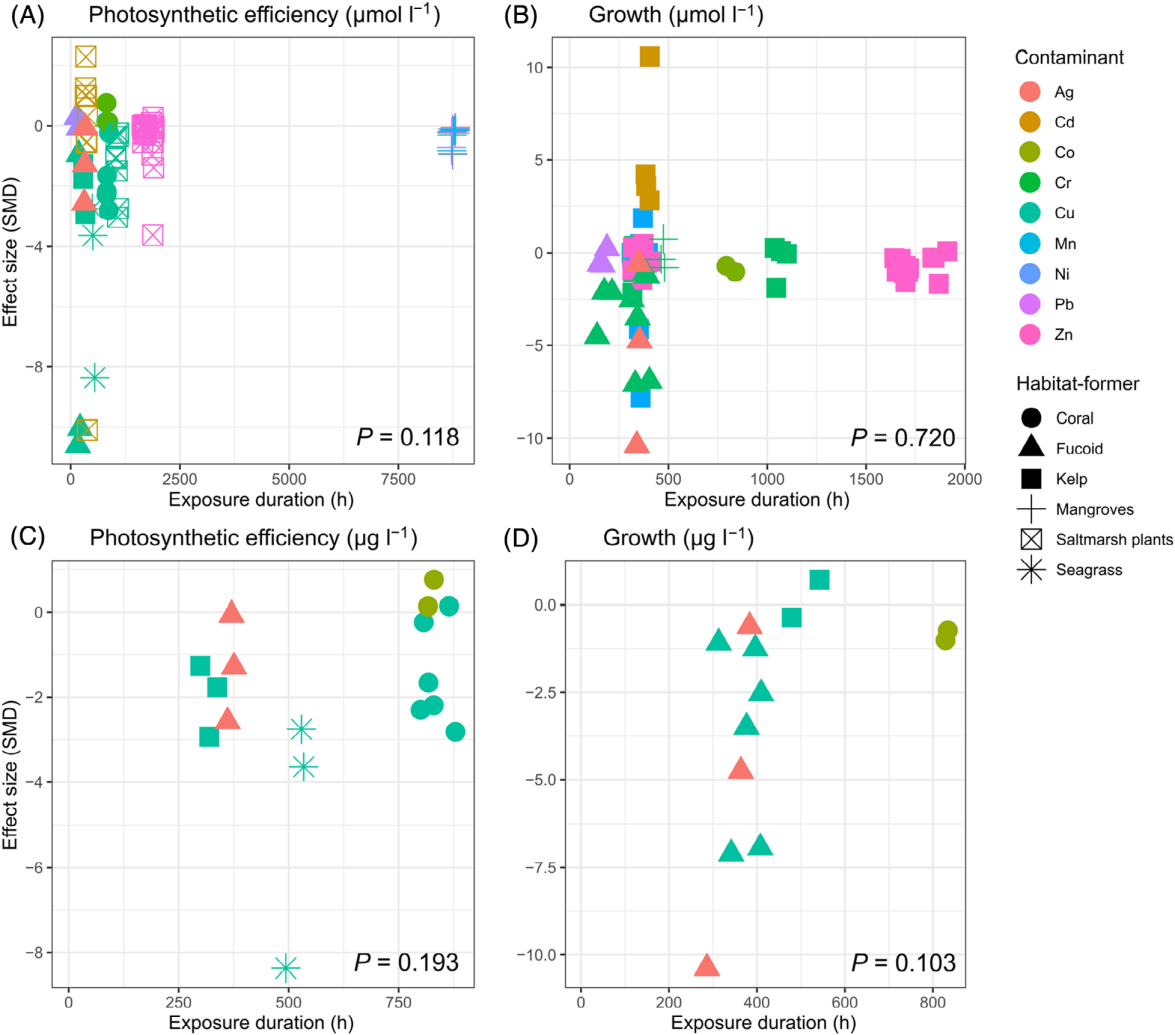
Meta‐regression of effect sizes (standardised mean difference; SMD) *versus* exposure time in (A, B) all studies and (C, D) studies that measured contamination in μg l^−1^. Data points show the tested contaminant (symbol colour) and habitat‐former (symbol shape). There were no significant relationships found between exposure duration and the variables shown.

There was no significant relationship between the duration of exposure of habitat‐formers to metals and effect sizes (Fig. 6). However, a lack of adequate replication across different types of habitat‐formers and metal concentrations, for example the type of habitat‐former may be a confounding factor, prevents us from generalising these results.

### (4) Qualitative analysis

#### (a) *Summary*

**Fig 7:**
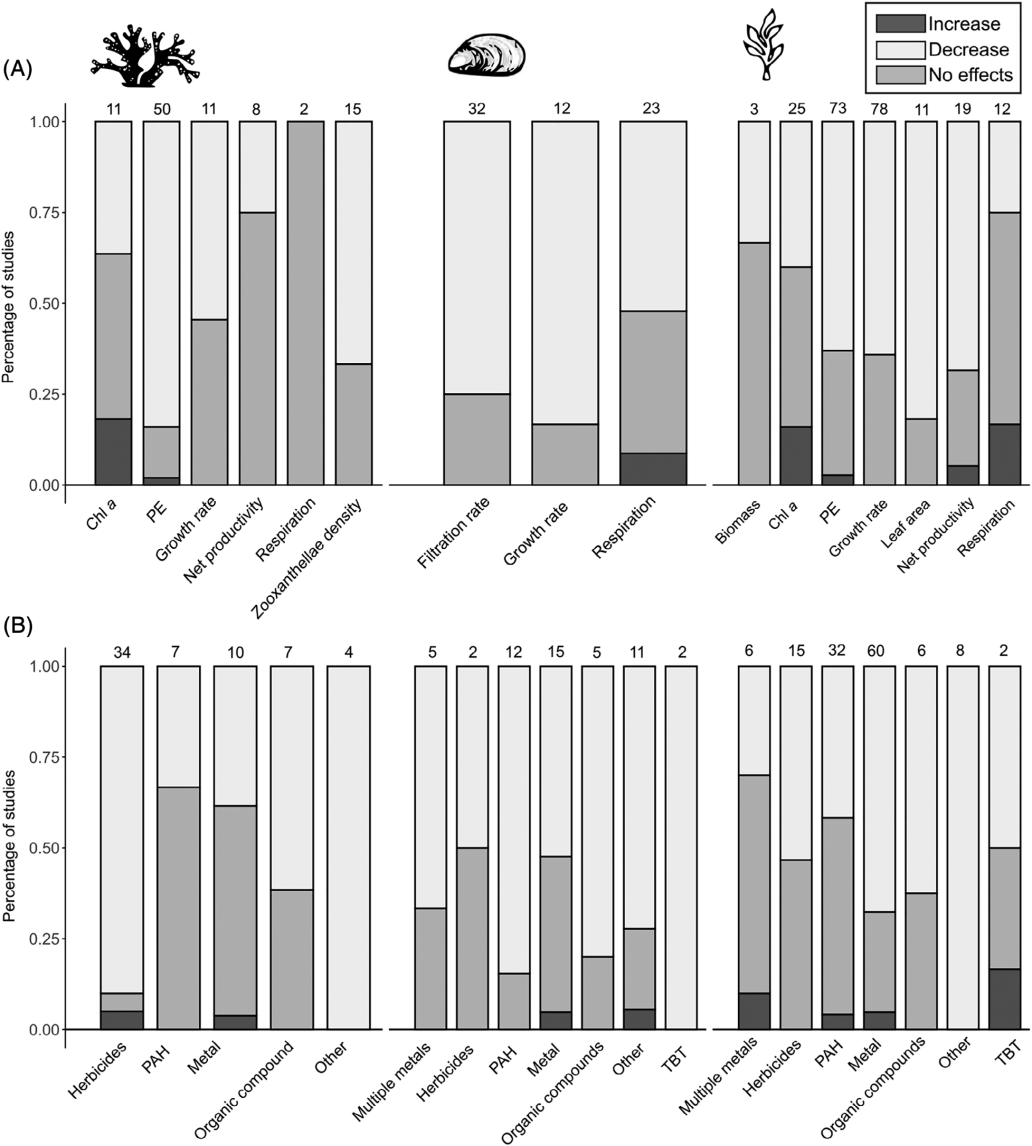
Percentage of studies included in the qualitative analysis showing an increase, decrease or no effect in responses in habitat‐forming corals (left), bivalves (centre), and plants and seaweeds (right) based on the function measured (A) or the contaminant (B). The number of studies within each category is shown above the bar. Chl *a*, chlorophyll *a*; PAH, polycyclic aromatic hydrocarbons; PE, photosynthetic efficiency; TBT, Tributyltin. Note that some studies in B measured more than one response variable.

The qualitative review showed an overall trend of negative impacts of contaminants in the functional attributes/performance of habitat‐formers (Fig. 7). Herbicides were the most studied contaminants on corals, while metals were more frequently studied in bivalves and plants and seaweeds (Fig. 7). In corals, the great majority of studies found negative effects of contaminants on photosynthetic efficiency as well as zooxanthellae density. In addition, six out of 11 studies on corals showed a decreased growth rate due to contamination (Fig. 7A). The most susceptible functional variable of plants and seaweeds to contamination seems to be leaf area of plants, with nine of 11 studies finding negative effects of toxicants. This was closely followed by net productivity (with 13 of 19 studies finding negative impacts) and growth rates and photosynthetic efficiency, with >60% of studies showing significant impacts of contaminants for each of these functional variables (50 out of 78 studies on growth; 46 out of 73 studies on photosynthetic efficiency). Most studies on bivalves found impacts of chemical contaminants on filtration (24 out of 32 studies) and growth rates (10 out of 12 studies) of these animals (Fig. 7A). However, 11 out of 23 studies (~48%) on respiration rates of bivalves found either no effects or increases in rates due to contamination (Fig. 7A).

#### (b) *Comparisons of results between qualitative and quantitative analyses*

Applying strict selection criteria for the studies included in the meta‐analyses, specifically with regard to the appropriateness of the methodology used, led to a significant reduction in the number of publications compared to the qualitative assessment. Sixty‐six per cent of studies that reported no effects of contaminants, on any given functional measure, were classified as having methodological flaws and were therefore excluded from the quantitative analyses. The biggest issue was a lack of appropriate replication (e.g. direct comparisons between one control *versus* one contaminated individual/site) or pseudo‐replication (Fig. S1). The habitat‐forming categories most impacted by methodological flaws, or for which the relevant data were not reported appropriately, were bivalves with ~60% of studies (31 out of 52 studies; 7 of which did not present data appropriately) and plants and seaweeds with 55% (71 out of a total of 129 studies, of which 15 did not present data appropriately; Fig. S1). Fifty per cent of studies of bivalves that measured filtration rates were categorised as containing methodological flaws (16 out of 32), and 39% of studies (9 out of 23) on bivalve respiration rates. All studies investigating the effects of contaminants on the respiration of plants and seaweeds contained some kind of methodological problem or did not present data in an appropriate way for meta‐analysis. Furthermore, many studies on the photosynthetic efficiency of plants and algae (59%; 38 out of 64) and their growth rate (37%; 29 out of 78), as well as those looking specifically at effects of herbicide (~53%; 27 out of 51 studies) were excluded from the quantitative analyses.

The largest differences between our qualitative assessment (Fig. 7) compared to the quantitative meta‐analyses were found in studies on corals. For example, although all studies that evaluated the effects of herbicides on the photosynthetic efficiency of corals found a significant negative impact, this pattern was not reflected in the quantitative meta‐analyses (Fig. 2; Table 1). Importantly, conclusions regarding the impacts of different contaminants differed in magnitude between the qualitative assessment (which included all available studies) and the quantitative meta‐analyses (which only included studies with appropriate design). For example, most studies that reported no effects of metals on corals were classified as poorly designed. Therefore, while 60% of all studies – regardless of design – found negative effects of metals on corals, this percentage increased to 80% when only considering studies with appropriate design. Similarly, 57.1% of all studies found negative effects of organic compound contamination on corals. However, this number increased to 80% when we disregarded poorly designed studies. This is the opposite pattern to what we would predict if publication bias was preferentially reporting significant impacts of contaminants. Of the 15 studies that evaluated the effects of herbicides on plants and seaweeds, only one was classified as appropriately designed, and reported negative effects.

## IV. DISCUSSION

Conservation practices are often based on the presence, absence or abundance of key species (Branton & Richardson, 2011). Similarly, assessments of the quality of habitats often focus on structural components of the habitat, such as abundance and composition of species, rather than functional parameters such as primary productivity (e.g. Fleishman *et al*., 2002; Culhane *et al*., 2014; Rubene *et al*., 2014). However, the direct translation of changes in the structural composition of an ecosystem into functional consequences is prone to error if we have no direct measures of performance (McMahon *et al*., 2012). This review and meta‐analysis revealed negative effects of multiple contaminants on important physiological traits of habitat‐formers, such as their photosynthetic efficiency, growth rates, leaf area and filtration rates. Our results suggest that chemical contaminants have the capacity directly to affect habitat quality and ecosystem functions that underpin critical ecosystem services. These findings can be used to direct future areas of study to fill important knowledge gaps to support the conservation of marine systems. Critically, this study reveals how sublethal effects of chemical contaminants have the potential to drive changes in key ecological functions of systems, highlighting the importance of considering such impacts in management and regulatory strategies.

Chemical contaminants, such as metals, have been linked to reduced growth rates of habitat‐forming species (Bryan, 1979; Amado Filho *et al*., 1997), reductions in the gross primary productivity of algae (e.g. Smith *et al*., 1984) and reduced filtration rates of bivalves (Vercauteren & Blust, 1999). Although such measures are intrinsically related to the functioning of biogenic habitats and, consequently, the services they provide, these effects are almost inevitably interpreted at the restricted scale of the individual (Browne *et al*., 2015). A general understanding of how particular sub‐lethal effects of chemical toxicants can affect the functioning and quality of habitats is, however, essential to establishing more efficient guidelines and practices for conserving biodiversity. In fact, one of the main aims of the European Union's Marine Strategy Framework Directive is to prevent impacts of contaminants at different levels of organisation (i.e. from individuals to whole systems). Our findings could usefully be incorporated into ecosystem models to predict contaminant effects on whole‐ecosystem functioning. However, to our knowledge, this has not yet been attempted for contaminants in marine systems dominated by one or a few key habitat‐forming species.

We found that contaminants at sub‐lethal concentrations reduced the photosynthetic efficiency of kelps and seagrasses, as well as the filtration of bivalves and growth rates of most of the studied habitat‐formers. In some studies, copper decreased filtration rates of oysters and mussels by up to 50% (Elfwing & Tedengren, 2002; Nicholson, 2003). Similarly, copper and lead reduced the photosynthetic activity of habitat‐forming producers by 40% or more (Connan & Stengel, 2011; Costa *et al*., 2016). Such effects have serious implications for ecosystem services and biological conservation. A decrease of 50% in the filtration power of oysters, for example, may have consequences almost as substantial as the actual loss of these organisms. Reductions in the ability of oysters to filter suspended particles from the water‐column potentially increases the likelihood of harmful algal blooms and can increase turbidity, which could consequently limit growth of seagrass (Beck *et al*., 2011). Nevertheless, current practices of monitoring are still based on abundance or presence of key species, including the use of surrogates or umbrella species (Caro & O'Doherty, 1999; Andelman & Fagan, 2000; Ormerod *et al*., 2010), and do not generally allow the detection of such impacts. The lack of visibility of sub‐lethal effects on function potentially delays remediation actions, aggravating impacts on the system (Sandin & Solimini, 2009).

Importantly, we demonstrate that sub‐lethal effects of contaminants on key physiological properties of habitat‐formers vary not only with the response being measured (e.g. growth or photosynthetic activity), but also with the identity of the habitat‐former species and the type of contaminant. This detailed information is of particular importance for the development of specific management and conservation guidelines. For example, we found that metals were the most widely damaging contaminant for habitat‐formers. In the absence of a more detailed study on a given system, restricting the input of metals and removing historical legacies of metal contamination might be considered a high priority from a conservation and management perspective (Hedge, Knott, & Johnston, 2009; Knott *et al*., 2009). Alternatively, where the main habitat‐former is seagrass, the effects of contamination by organic compounds on photosynthetic efficiency should be taken into account when prioritising management actions.

We did not find any conclusive relationships between duration of exposure to metals and effect sizes. This could be due, however, to the low numbers of studies across the different types of habitat‐formers and/or concentrations of metals. Moreover, the duration of exposure to contaminants, or of the studies investigating this exposure, might not have been long enough to observe significant effects. Further experiments with appropriate temporal replication and durations are needed to investigate this issue. Similarly, we found few significant relationships between concentrations of contaminants (metals), and the magnitude of their impacts on the key functional attributes of habitat‐forming species. The relationships observed here, when significant, varied with the type of metal and habitat‐former. Predictions of impacts based solely on concentrations of single contaminants found in natural habitats are thus highly unlikely to be precise and may vary depending on duration of exposure. In some of the studies designed to measure the full range of sublethal effects on habitat‐formers, some of the concentrations employed are unrealistically high, and perhaps not ‘environmentally relevant’. Our meta‐regression using a more restricted range of concentrations (up to 500 μg l^−1^) gave a different pattern in the results for relationships between metal concentration and effect size for photosynthetic efficiency. The rapid pace and spread of chemical contaminants worldwide, the increasing reliance of daily human activities on these chemicals (Bernhardt *et al*., 2017) and the multiplying anthropogenic pressures affecting marine systems across the globe (Crain, Kroeker, & Halpern, 2008; Halpern *et al*., 2008) are likely to affect our sense of what is environmentally relevant. Contaminants are now being found in high concentrations even in remote parts of the planet. Jamieson *et al*. (2017), for instance, found high concentrations of polychlorinated biphenyls (PCBs) and polybrominated diphenyl ethers (PBDEs) in amphipods from two of the deepest ocean trenches (>10000 m deep). The concentrations found were 50 times greater than in crabs from a highly polluted river system in China, suggesting that the delivery of these pollutants occurs over long distances despite regulation since the 1970s (Dafforn, 2017). In Lake Macquarie, NSW, Australia, the largest coastal saltwater lagoon in the southern hemisphere, metal concentrations in surface water samples from sites with the seagrass *Zostera capricorni* reached 570, 62, 370 and 66 μg l^−1^ for Zn, Cd, Pb and Cu, respectively (Batley, 1987). Importantly, most coastal systems worldwide are increasingly exposed to multiple chemical contaminants, the combination of which can cause unexpected impacts on the diversity and functioning of communities (O'Brien *et al*., 2019).

Climate change is expected to have complex interactions with chemical contaminants, altering not only the frequency and rate at which they enter coastal systems (IPCC, 2014), but also the potential impacts of contaminant cocktails through changes in their toxicity as well as in the susceptibility/tolerance of organisms (Noyes *et al*., 2009). The toxicity of metals and pesticides, for instance, has been shown to increase with rising temperature (Bryan, 1971; McLusky *et al*., 1986; Boone & Bridges, 1999), which is also likely to enhance the rate of uptake of contaminants due to increased metabolic rates and decreased oxygen solubility (e.g. Kennedy, Gill, & Walsh, 1989; Noyes *et al*., 2009). The patterns observed in our systematic review provide a window into a rapidly changing world and should be used as clear evidence that chemical contaminants have the potential to alter the functioning of systems through sublethal effects on habitat‐forming species.

The impact of contaminants on the performance of key species presents a strong argument for using functional endpoints in toxicity testing, ecological risk assessments and conservation planning. The use of functional endpoints may be a more relevant and potentially more sensitive, efficient and cost‐effective method of ecological risk assessment than simply measuring the abundance of species or the lethal concentration (e.g. LC_50_) for selected species (Johnston *et al*., 2015). Moreover, endpoints that can be directly linked to ecosystem functions are a more direct measure of ecosystem disruption than biochemical or cellular markers of biotic injury in habitat‐forming species (e.g. see Edge *et al*., 2014). Although herein we restricted our examination to direct, sublethal effects of contaminants on functional properties of key species, such impacts are likely to feed forward to populations of other species and to communities *via* indirect mechanisms even after elimination of the original source(s) of contamination. For example, changes in the diversity and abundance of epifaunal communities in seagrass meadows contaminated with metals have been attributed to a reduction in seagrass epiphyte palatability due to toxicant accumulation (Marín‐Guirao *et al*., 2005). The contaminant‐driven changes in the filtration rates of bivalves shown here could lead to overall changes in nutrient cycling in the habitats they occupy (Beck *et al*., 2011), resulting, for example, in decreased water quality with direct consequences to a whole suite of local species, such as impacts on the growth of seagrasses and harmful algal blooms. Therefore, changes in the functional performance of one type of habitat‐former can potentially have negative consequences on the functionality of other habitat‐forming species.

This review and meta‐analysis highlights some important gaps in knowledge that need to be filled if we are to manage and conserve effectively these key species and the habitats they form. Metals are, by far, the most studied chemical contaminant, even though the production, application and diversification of synthetic chemicals, such as pesticides and pharmaceuticals, have likely outpaced more recent use of metals (e.g. Bernhardt *et al*., 2017). Moreover, while the authors recognise that establishing field experiments in marine environments can be logistically and financially demanding, which may influence the experimental design, replication at an appropriate scale remains necessary to test hypotheses (see extensive discussion in Mayer‐Pinto *et al*., 2010). How a study is designed can influence the results obtained and their interpretation and consequently, management priorities based on those findings. We found that including poorly designed studies in our assessment would have led to underestimates of the effects of contaminants on habitat‐formers, with important implications for management. Additionally, most of our current knowledge on the effects of contaminants is derived from laboratory studies. Laboratory experiments are useful for establishing toxicant guidelines, but are limited in that they may not reflect the complex conditions of natural systems, such as interactions among and within species and the physical dynamics of the system (Connell, 1974; Underwood & Peterson, 1988). Interactions between contamination and ecological processes such as competition, for example, could modify the responses of organisms to a toxicant (Johnston & Keough, 2003). In addition, laboratory studies are usually performed on at most a few individuals, so any effects observed would not reflect potential large‐scale impacts in areas with large aggregations of individuals, which is often the case for habitat‐formers. Moreover, when a contaminant is released in the field, many factors could influence its toxicity (McLusky *et al*., 1986; Schiedek *et al*., 2007), which may be absent in the laboratory. Additional experiments should aim to include more complex and relevant scenarios to increase our ability correctly to predict effects of contaminants on natural ecosystems (Browne *et al*., 2016).

Gaps in the published literature limit our potential to create marine contaminant guidelines that protect ecosystem function. Importantly, we hope that this review and meta‐analyses will be considered as a guide to future research. We show that contamination by chemical toxicants has the potential to affect ecosystem functioning and habitat quality through sub‐lethal impacts on habitat‐forming organisms. We emphasise, however, the need for studies that explore the functional consequences of sub‐lethal effects at the scale of habitats and ecosystems. We also highlight the need to incorporate the use of functional endpoints into monitoring and conservation studies. In the interim, our findings could be incorporated into ecosystem models that attempt to predict the direct effects of contaminants on the functioning of marine ecosystems.

## V. CONCLUSIONS

1. Our global systematic review and meta‐analysis demonstrates links between effects of contaminants at lower levels of organisation (i.e. at the biochemical and/or physiological level of individuals) to ecological, large‐scale impacts, through sub‐lethal effects on the functioning of habitat‐forming species.
2. Negative effects of contaminants were observed on the functionality of all habitat‐forming groups (corals, seagrass, bivalves etc.). The magnitude of impacts by contaminants is likely to vary with contaminant concentration, type of habitat‐forming organism and the functional property being measured.
3. Metals had widespread negative effects on physiological functions of habitat‐formers. However, copper was by far the most studied metal, so future well‐designed studies on a wider range of metals (and their combinations) will be important to increasing our understanding of the overall impacts of this class of contaminants.
4. A critical evaluation of the quality of the available studies revealed a large number with design flaws. A greater proportion of studies without these design flaws found negative effects of contaminants on habitat formers. We encourage researchers to think carefully about experimental design and the treatments (including controls) that are most appropriate to test unambiguously for the effects of contaminants on individuals, populations and/or habitats.
5. We hope that this review will guide future research and inform the development of conservation and management models, policies and practices tailored to specific habitats and types of contamination. We strongly recommend the consideration of sub‐lethal effects of contaminants as a tangible threat to ecosystem structure and functioning.

## Supporting information


**Fig. S1.** Proportions of studies assessed as having either appropriate design, control problems, replication problems, both control and replication problems, or unclear design.

**Table S1.** List of the publications systematically reviewed for the qualitative analysis.

**Table S2.** Summary of data taken from publications used in the meta‐analysis.
