# Supplementary figures and images for: Sublethal effects of contaminants on marine habitat‐forming species: a review and meta‐analysis
Source: Biol Rev Camb Philos Soc. 2020 Jul 2;95(6):1554–73. doi: 10.1111/brv.12630 (PMC7689725; doi:10.1111/brv.12630)

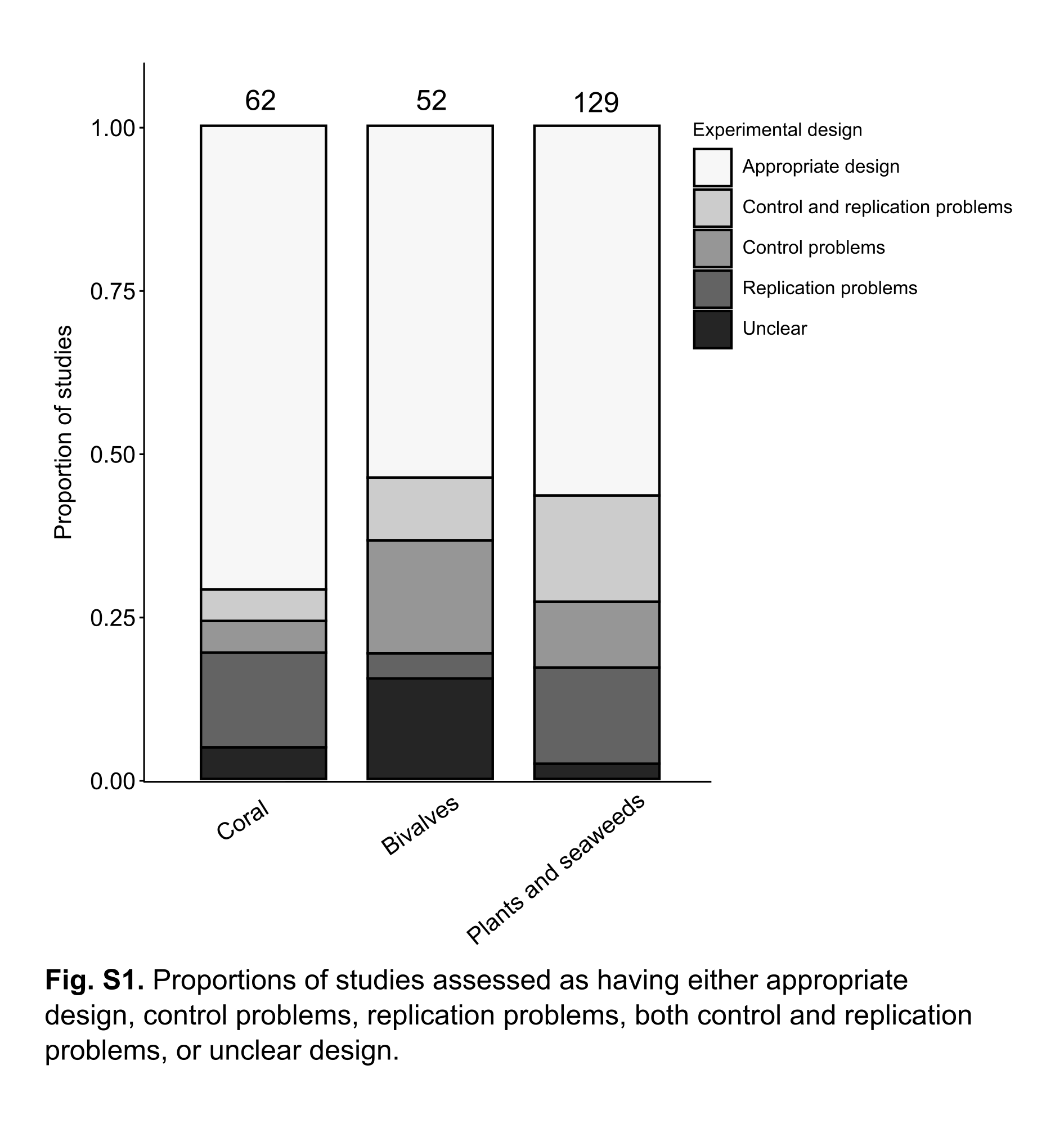

Supplement: Supplementary file 1 — Fig. S1. Proportions of studies assessed as having either appropriate design, control problems, replication problems, both control and replication problems, or unclear design. [file BRV-95-1554-s001.png]
