# Supplementary material for: Sublethal effects of contaminants on marine habitat‐forming species: a review and meta‐analysis
Source: Biol Rev Camb Philos Soc. 2020 Jul 2;95(6):1554–73. doi: 10.1111/brv.12630 (PMC7689725; doi:10.1111/brv.12630)
Supplement: Supplementary file 2 — Table S1. List of the publications systematically reviewed for the qualitative analysis. [file BRV-95-1554-s002.docx]

**Table S1.** List of the publications systematically reviewed for the qualitative analyses. The column headed Meta-analysis identifies the subset of studies that were included in the quantitative meta-analysis, with reasons for exclusion of papers that did not meet the selection criteria listed in Section II.2.

| **Authors** | **Meta-analysis** | **Comments** |
| --- | --- | --- |
| Abel (1976) | N | Problem with controls and results are not reported appropriately for data extraction |
| Al-Subiai *et al.* (2011) | Y |  |
| Alutoin *et al.* (2001) | N | Problems with replication |
| Andrades-Moreno *et al.* (2013) | Y |  |
| Antrim *et al.* (1995) | N | Results are not reported appropriately for data extraction |
| Avolizi & Nuwayhid (1974) | N | Unclear methodology and results are not reported appropriately for data extraction |
| Azarbad *et al.* (2010) | N | Problems with controls |
| Batley *et al.* (1992) | N | Results are not reported appropriately for data extraction |
| Baumann *et al.* (2009) | N | Problems with replication and results are not reported appropriately for data extraction |
| Beiras *et al.* (2012) | N | Unclear methodology and results are not reported appropriately for data extraction |
| Bielmyer *et al.* (2010) | Y |  |
| Biscere *et al.* (2015) | Y |  |
| Blackmore & Wang (2003) | N | Problems with controls and replication |
| Brinza *et al.* (2009) | N | Results are not reported appropriately for data extraction |
| Cantin *et al.* (2007) | Y |  |
| Cambrollé *et al.* (2012a) | Y |  |
| Cambrollé *et al.* (2012b) | Y |  |
| Cambrollé *et al.* (2016) | Y |  |
| Chen *et al.* (2012) | N | Problems with controls and results are not reported appropriately for meta-analysis |
| Connan & Stengel (2011) | Y |  |
| Costa *et al.* (2016) | Y |  |
| Culbertson *et al.* (2008) | N | Problems with controls |
| Downs *et al.* (2014) | N | Results are not reported appropriately for data extraction |
| dos Santos *et al.* (2015) | Y |  |
| Elfwing & Tedengren (2002) | Y |  |
| Flores *et al.* (2013) | N | Problems with controls and replication |
| Gao *et al.* (2011) | Y |  |
| Grant *et al.* (2003) | N | Problems with controls and replication |
| Harrington *et al.* (2005) | N | Problems with replication and results are not reported appropriately for data extraction |
| Hermsen *et al.* (1994) | Y |  |
| Hsiao *et al.* (1978) | N | Results are not reported appropriately for data extraction |
| Hu & Wenjiao (2015) | Y |  |
| Huovinen *et al.* (2010) | Y |  |
| James *et al.* (1987) | N | Problems with controls |
| Jensen *et al.* (2004) | N | Problems with control and replication and results are not reported appropriately for data extraction |
| Jones & Kerswell (2003) | Y |  |
| Jones *et al.* (1999) | Y |  |
| Jones & Heyward (2003) | N | Results are not reported appropriately for data extraction |
| Jones *et al.* (2003) | Y |  |
| Jorge *et al.* (2007) | Y |  |
| Kwok *et al.* (2016) | N | Results are not reported appropriately for data extraction |
| Kegler *et al.* (2015) | Y |  |
| Lin *et al.* (2002) | N | Results are not reported appropriately for data extraction |
| Linden *et al.* (1987) | N | Results are not reported appropriately for data extraction |
| Llagostera *et al.* (2016) | Y |  |
| Macinnis-Ng & Ralph (2002) | N | Problems with replication |
| Macinnis-Ng & Ralph (2003) | N | Results are not reported appropriately for data extraction |
| Manley (1983) | N | Problems with controls |
| Márquez-García *et al.* (2013) | Y |  |
| Martin *et al.* (2015) | Y |  |
| McMahon *et al.* (2005) | N | Problems with controls |
| Mercurio *et al.* (2004) | Y |  |
| Montenegro *et al.* (2016) | Y |  |
| Naidoo *et al.* (2014) | Y |  |
| Negri *et al.* (2005) | N | Results are not reported appropriately for data extraction |
| Negri *et al.* (2011) | N | Problems with controls and results are not reported appropriately for data extraction |
| Nell & Livanos (1988) | Y |  |
| Nguyen *et al.* (2017) | Y |  |
| Nicholson (2003) | Y |  |
| Ostroumov & Widdows (2006) | N | Results are not reported appropriately for data extraction |
| Owen *et al.* (2002) | N | Results are not reported appropriately for data extraction |
| Pan *et al.* (2016) | N | Results are not reported appropriately for data extraction |
| Peckol *et al.* (1990) | N | Problems with controls |
| Poulsen *et al.* (1982) | N | Problems with controls and replication and results are not reported appropriately for data extraction |
| Price *et al.* (1986) | Y |  |
| Rahman *et al.* (2012) | Y |  |
| Ramesh *et al.* (2015) | Y |  |
| Redondo-Gómez *et al.* (2014) | Y |  |
| Reinert *et al.* (2016) | N | Results are not reported appropriately for data extraction |
| Richter *et al.* (2016) | Y |  |
| Rocha *et al.* (2014) | Y |  |
| Rosemarin *et al.* (1994) | N | Problems with controls and replication and results are not reported appropriately for data extraction |
| Santos *et al.* (2015) | Y |  |
| Sabourin & Tullis (1981) | N | Unclear methodology and results are not reported appropriately for data extraction |
| Scarlett *et al.* (2005) | Y |  |
| Scarlett *et al.* (2011) | N | Unclear methodology and results are not reported appropriately for data extraction |
| Shafir *et al.* (2003) | N | Unclear methodology and results are not reported appropriately for data extraction |
| Shaw *et al.* (2008) | N | Results are not reported appropriately for data extraction |
| Smith *et al.* (1984) | Y |  |
| Sodré *et al.* (2013) | Y |  |
| Tarrant *et al.* (2004) | Y |  |
| Tedengren & Kautsky (1987) | Y |  |
| Thomas *et al.* (1999) | N | Problems with controls |
| Valkirs *et al.* (1987) | N | Problems with replication and results are not reported appropriately for data extraction |
| Vercauteren & Blust (1999) | Y |  |
| Wall *et al.* (2001) | N | Problems with controls |
| Walsh *et al.* (1982) | N | Problems with replication and results are not reported appropriately for data extraction |
| Wang *et al.* (2005) | N | Problems with controls and replication |
| Wang *et al.* (2012) | N | Problems with controls and replication and results are not reported appropriately for data extraction |
| Wang *et al.* (2014) | Y |  |
| Watanabe *et al.* (2006) | N | Results are not reported appropriately for data extraction |
| Wilson *et al.* (2004) | N | Problems with controls |
| Wrabel & Peckol (2000) | Y |  |
| Wu *et al.* (2015) | Y |  |
| Zhu *et al.* (2015) | N | Problem with controls and results are not reported appropriately for data extraction |
